# Schizophyllan Optimization and Production in Submerged Cultures of Different *Schizophyllum commune* Isolates Collected in Thailand

**DOI:** 10.3390/jof12050321

**Published:** 2026-04-28

**Authors:** Soravit Chaimongkol, Wittayothin Yingkulchao, Nattawut Rungjindamai, Nguyen Tai Toan, Borworn Werapan, Kwanruthai Malairuang, Phongsawat Khamsuntorn, Sayanh Somrithipol, Somjit Komwijit, Sujinda Sommai, Umpawa Pinruan, Wai Prathumpai

**Affiliations:** 1 Department of Biology, School of Science, King Mongkut’s Institute of Technology Ladkrabang, Bangkok 10520, Thailand; soravit.kmitl@gmail.com (S.C.); wittayothin@gmail.com (W.Y.); 2 National Center for Biotechnology and Genetic Engineering (BIOTEC), National Science and Technology Development Agency (NSTDA), 111 Thailand Science Park, Phahonyothin Road, Khlong Nueng, Khlong Luang, Pathum Thani 12120, Thailand; borworn.we@gmail.com (B.W.); kwanruthaimalai@gmail.com (K.M.); phongsawat.kha@ncr.nstda.or.th (P.K.); sayanh@biotec.or.th (S.S.); somjit.kom@biotec.or.th (S.K.); sujinda.som@biotec.or.th (S.S.); umpawa.pin@biotec.or.th (U.P.); 3 School of Agriculture and Natural Resource, Vinh University, Truong Vinh 46000, Nghe An, Vietnam; toannt@vinhuni.edu.vn

**Keywords:** bioreactor, central composite design, Plackett–Burman design, schizophyllan, *Schizophyllum commune*

## Abstract

Twenty strains of *Schizophyllum commune* from the BIOTEC culture collection were selected for this study. *S. commune* is characterized by white to gray fan-shaped caps with lobed margins and distinctive split gills. Phylogenetic analysis of combined LSU rDNA and ITS rDNA sequences data using maximum parsimony placed the fungi in a strongly supported clade with *S. commune*. All strains were primarily screened for exopolysaccharide (EPS) and biomass production using potato dextrose broth (PDB) and peptone yeast glucose medium (PYGM) in 250 mL flasks shaken at 200 rpm for 7 days. The results revealed three strains with high EPS production, each exceeding 2.3 g/L, namely MMCR00487, MMCR00474 and MMCR00256. These strains were selected for media optimization using a Plackett–Burman design. Among them, MMCR00256 exhibited the highest EPS yield of 8.34 ± 1.47 g/L, followed by MMCR00487 and MMCR00474. Therefore, the strain MMCR00256 was further optimized by central composite design. The results revealed that the optimized medium for MMCR00256 increased the production of EPS to 10.39 ± 1.69 g/L, with a biomass yield of 26.28 ± 1.63 g/L (395 mg/g). The 5 L bioreactor optimization tested two inoculum types (mycelial and pellet) and two media (CCD and estimated) using strain MMCR00256. The mycelial inoculum grown in the estimated medium produced the highest EPS yield of 8.37 ± 0.26 g/L after 3 days, with 13.56 ± 2.94 g/L biomass. In conclusion, this study demonstrates that *S. commune* MMCR00256, when cultivated using the estimated medium and mycelial inoculum, can achieve enhanced exopolysaccharide production with improved efficiency, highlighting its significant potential for the development of efficient and scalable schizophyllan production processes at the industrial scale. Furthermore, this study provides essential insights into the cultivation and optimization of schizophyllan in *S. commune*.

## 1. Introduction

Schizophyllan is a polysaccharide produced by the fungus *Schizophyllum commune* and is composed of β-glucans, with a main backbone consisting of glucose molecules (D-glucose) linked between carbon atoms 1 and 3 in a long chain [1]. Schizophyllan is also produced by other fungi, such as *Athelia rolfsii* [2]. Furthermore, schizophyllan is known by various names, such as sonifilan, sizofiran and sizofilan. Schizophyllan is a high-value product that is widely used as a supplement in food, pharmaceutical and cosmetic products, with numerous studies highlighting its anticancer, antitumor and immunomodulatory properties [3,4]. Over the past five years, schizophyllan has been extensively reviewed, both in general [5,6] and with a focus on its biomedical and pharmaceutical applications. Recent studies have demonstrated its potential in wound healing [7,8,9], lipase inhibitory activity associated with anti-obesity and anti-lipidemic effects [10], and regulation of osteoclast and osteoblast differentiation, suggesting its therapeutic potential for bone-related diseases [11]. In addition, schizophyllan-based hydrogels have gained considerable attention [7,12,13], while its anti-photoaging and melanin-inhibitory properties highlight its value in dermatological and cosmetic applications [14]. Parallel to these applications, ongoing research has emphasized production optimization, including the use of additives to enhance yield [15,16] and circular bioeconomy approaches utilizing industrial by-products such as brewer’s spent grain [17]. Furthermore, the benefits of schizophyllan have also been explored in economically important aquatic species such as whiteleg shrimp [18]. *Schizophyllum commune* is a dominant species within this genus due to its frequent occurrence. However, two other closely related species in this genus, namely *S. radiatum* and *S. umbrinum*, were identified in the present study. These findings represent the first record of these species in Thailand. According to Carreño-Ruiz [19] these three species are widely found in tropical regions. Additionally, *S. commune* is documented as an opportunistic pathogen associated with respiratory tract infections [20]. With respect to the cultivation of *Schizophyllum* species and the production of schizophyllan, Kumari and Survase [21] reported favorable conditions. Their study revealed that sugars, including sucrose and glucose, were beneficial for schizophyllan production. Among organic nitrogen sources, beef extract and yeast extract were found to be effective, while ammonium nitrate was identified as a suitable inorganic nitrogen source. The objectives of this study were to screen the ability of strains of *S. commune* collected in Thailand to produce schizophyllan, to optimize these strains for the production of schizophyllan in liquid culture, and to evaluate the ability of the strain with the most potential to produce schizophyllan in a bioreactor.

## 2. Materials and Methods

### 2.1. Fungal Culture and Maintenance

A total of 20 strains of *S. commune* were collected and isolated from different habitats in Thailand (Table 1) between November 2012 and February 2021 by the Plant–Microbe Interactions Research Team of the National Center for Genetic Engineering and Biotechnology (BIOTEC), Pathum Thani, Thailand. All the strains were identified through a morphological study and 18S rRNA gene sequence analysis. The fungal cultures were deposited at the BIOTEC Culture Collection (BCC) on potato dextrose agar (PDA, Difco™, Becton, Dickinson and Company, Franklin Lakes, NJ, USA) and stored at −80 °C (Thermo Fisher Scientific, REVCO ULT-1786-7V, Asheville, NC, USA) in cryotubes containing 10% glycerol for further studies.

### 2.2. Morphology

The morphological characteristics of the *Schizophyllum* species studied were documented through field observations. The sizes of the largest and smallest mushroom caps were measured using a ruler. Photographs were taken, along with detailed notes on features such as the cap, gills, stipe, flesh and color using a standard color guide (Munsell Color Services, Grand Rapids, MI, USA) [22]. After the external features were examined, the specimens were sectioned and stained with Congo red mixed with 5% potassium hydroxide [19]. The stained specimens were then observed under a microscope (Olympus, CX31RBSFA, Tokyo, Japan) to study internal cellular structures, including basidiospores, basidia, cystidia, hyphae, and clamp connections [19]. Measurements and photographs were taken to prepare the plates for further analysis.

### 2.3. Molecular Phylogeny

The strains used in the study are listed in Table 1. Genomic DNA was extracted using the CTAB method [23]. Two gene regions, the internal transcribed spacer (ITS) and the large subunit (LSU), were amplified through polymerase chain reaction (PCR). The ITS region was amplified using the primers ITS1F (5′-CTTGGTCATTTAGAGGAAGTAA-3′), ITS5 (5′-GGAAGTAAAAGTCGTAACAAGG-3′), and ITS4 (5′– TCCTCCGCTTATTGATATGC-3′) [24], while the LSU region was amplified using the primers LROR (5′–ACCCGCTGAACTTAAGC-3′) and LR5 (5′-TCCTGAGGGAAACTTCG-3′). The PCR conditions for ITS amplification were as follows: initial denaturation at 96 °C for 2 min 35 amplification cycles with annealing at 53 °C for 1 min and a final extension at 72 °C for 7 min. For LSU amplification the PCR conditions consisted of initial denaturation at 95 °C for 2 min 34 amplification cycles with annealing at 55 °C for 1.30 min and a final extension at 72 °C for 10 min [25]. PCR products were sent to Macrogen, Inc., South Korea, for DNA sequencing analysis. DNA sequences were analyzed and compared with those from the GenBank database and aligned using Geneious v.9.1.4 [26]. Sequence analysis was conducted using MAFFT version 7. A phylogenetic tree was inferred by maximum parsimony (MP) analysis with PAUP* 4.0b10, incorporating 10 replicates of stepwise additions, heuristic searches, and 1000 random taxa. The robustness of the most parsimonious tree was evaluated using 1000 bootstrap replications.

### 2.4. Seed Culture Preparation

The seed culture preparation was divided into three types: mycelia (A), large pellets (B) and small pellets (C). The mycelial seed culture (A) was prepared by first cultivating *Schizophyllum* sp. on PDA at 25 °C for 7 days. Small pieces (1 cm^2^) were cut from the agar culture using a sterile scalpel and transferred into 250 mL flasks containing 50 mL of potato dextrose broth (PDB; Difco™, Becton, Dickinson and Company, Franklin Lakes, NJ, USA), which had been sterilized at 121 °C for 15 min. The liquid seed cultures were incubated at 200 rpm for 5 days at 25 °C on a rotary shaker (New Brunswick, NJ, USA). The culture was then blended in a sterile blender (Waring, 7010S 1L two-speed blender, Waring Commercial, Torrington, CT, USA) to prepare the inoculum starter. The large pellet seed culture (B) was prepared following the same procedure as the mycelial culture but without the blending step. The small pellet seed culture (C) was prepared by transferring the mycelia into PDB and shaking at 200 rpm for 2 days at 25 °C to form small pellets.

### 2.5. Secondary Screening of Exopolysaccharide by S. commune

For secondary screening, a 10% (*v*/*v*) large pellet seed culture was used as the inoculum for secondary screening because it provides a standardized morphological form that allows consistent comparison of EPS production among multiple isolates. Large pellet seed culture of 20 strains of *S. commune* was transferred into 250 mL flasks containing 50 mL of PDB (standard medium) (20 g/L dextrose and 4 g/L potato starch) and 250 mL flasks containing 50 mL of peptone yeast extract medium (PYGM) (20 g/L yeast extract, 10 g/L glucose, 5 g/L peptone, 1 g/L KH_2_PO_4_ and 0.5 g/L MgSO_4_•7H_2_O) in triplicate. The flasks were incubated at 25 °C on a rotary shaker at 200 rpm for 7 days. The culture broths were then vacuum filtered through Whatman^®^ No. 1 filter paper (Maidstone, UK) to separate the mycelia. The liquid culture broth was then mixed with 95% cold ethanol (4:1) (four parts ethanol to one part supernatant) for exopolysaccharide precipitation, which was incubated at −20 °C overnight and lyophilized. Mycelia on preweighed filter papers were oven-dried at 80 °C for 3 days to obtain dry weights. *S. commune* isolates that gave EPS concentrations higher than 2.30 g/L were selected for further medium optimization using the Plackett–Burman design (PBD) and the central composite design (CCD).

### 2.6. Optimization of Exopolysaccharide by Selected Fungi

#### 2.6.1. Optimization of Exopolysaccharide by *S. commune* Using the Plackett–Burman Design in 250 mL Flasks

A Plackett–Burman design was used, and 11 factors (factors A–K) (Table 2) were selected to generate 26 treatments (Table 3) for assessing the impact of growth medium factors on exopolysaccharide production by the 3 selected strains of *S. commune*. PDB and PYGM were used as controls. A 10% (*v*/*v*) mycelial inoculum of each selected mushroom strain was transferred to 250 mL flasks containing 50 mL of different liquid media. The liquid culture was incubated at 25 °C on a rotary shaker at an agitation speed of 200 rpm for 7 days. All experiments were carried out in triplicate. Biomass and exopolysaccharide were measured, and three selected strains of *S. commune* that produced the greatest amount of exopolysaccharide were selected for optimization using the central composite design.

#### 2.6.2. Optimization of Exopolysaccharide Production by *S. commune* Using Central Composite Design in 250 mL Flasks

The highest level of exopolysaccharide production by *S. commune* was detected using the Plackett–Burman (PB) design, and the nutritional optimization of these plants was analyzed using the central composite design. Experimental design and statistical analysis were performed using Design-Expert software (Version 13; Stat-Ease Inc., Minneapolis, MN, USA). PDB and PYGM were used as controls. A 10% (*v*/*v*) mycelial inoculum of the selected potential strain was transferred to 250 mL flasks containing 50 mL of different liquid media. The liquid culture was incubated at 25 °C on a rotary shaker at 200 rpm for 7 days. All experiments were carried out in triplicate. Afterward, the biomass and exopolysaccharide content of the fermentation liquid in the flask were measured (details described in Section 2.8.1 and Section 2.8.2, respectively). The estimated and optimized medium composition will be analyzed and obtained using Design-Expert software based on the maximum exopolysaccharide production. *S. commune* and the liquid treatment that yielded the highest exopolysaccharide concentration were selected for assessing exopolysaccharide in the chosen mushroom strain using a 5 L bioreactor in the next step.

### 2.7. Evaluation of Exopolysaccharide Production in a 5 L Bioreactor

The selected strain of *S. commune* was cultivated in a 5 L bioreactor (Biostat^®^ B, Sartorius Stedim Biotech, Göttingen, Germany) with a working volume of 4 L. The optimized medium was autoclaved at 121 °C for 15 min, while glucose was sterilized separately and added aseptically to the bioreactor. First, the inoculum was prepared in PDB as described in Section 2.4, and 10% (*v*/*v*) inoculum was added to the bioreactor. To evaluate the effect of inoculum type on exopolysaccharide at the bioreactor scale, two optimized inoculum forms were tested: a mycelial inoculum and a small pellet inoculum. Each inoculum type was prepared following previously described procedures. Two growth medium formulations were studied for comparison: the CCD formulation from a previous study and the estimated formulation (Table 4). Cultivation was carried out at 25 °C with an air supply of 1 vvm. The impeller speed was maintained at 100 rpm for the first 2 days and then increased to 300 rpm for the remaining 5 days. Samples were withdrawn daily to determine the dry weight, dry weight of the exopolysaccharide and residual glucose concentration.

### 2.8. Analysis of the Exopolysaccharide

#### 2.8.1. Biomass Determination

The whole culture broth of *S. commune* was centrifuged (Avanti JXN-26, Beckman Coulter, Brea, CA, USA) at 14,000 rpm for 15 min at 4 °C. The supernatant was collected and filtered through Whatman No. 1 filter paper to separate the exopolysaccharide (in the supernatant) and the mycelia (in the pellet). The mycelia were subsequently washed with sterile distilled water on filter paper and dried at 70 °C for 72 h until a constant weight was achieved. The culture filtrate was subjected to exopolysaccharide purification (Section 2.8.2).

#### 2.8.2. Exopolysaccharide Purification

The supernatant of the centrifuged broth from the biomass determination step was mixed with 95% ethanol and stored at 4 °C for 12 h for exopolysaccharide precipitation. During ethanol precipitation, the exopolysaccharide polymer aggregates and floats to the upper phase, whereas monosaccharides and dissolved salts remain in the lower phase. The polymer solution was then dialyzed with a Cellusep 20 kDa cut-off dialysis membrane in a 4-volume distilled water to diminish leftover salts in the solution. Afterward, the polymer was freeze-dried (Telstar, LyoSlfa) at −80 °C for 5 days to achieve a constant weight.

#### 2.8.3. Molecular Weight Determination by Gel Permeation Chromatography (GPC)

The exopolysaccharide was dissolved at a concentration of 2 mg/mL in 0.05 M sodium bicarbonate buffer (pH of 11.0) and filtered through a 0.45 μm membrane filter before injection. Molecular weight analysis was performed on a Waters 600E GPC system using a PL aquagel-OH MIXED-H 8 µm column (Agilent). Deionized water was used as the mobile phase at a flow rate of 0.5 mL/min and the column temperature was maintained at 80 °C. A refractive index detector was used for the detection of eluted molecules. Dextran (MW 4400–401,000) was used as a standard.

#### 2.8.4. Measurement of Exopolysaccharide Viscosity

Approximately 1–2 g of exopolysaccharide was finely ground and dissolved in distilled water to prepare the test solution. The viscosity was measured with Viscometer RVDV II+ (Brookfield, WI, USA) and reported in centipoise (cP). These measurements indicated the estimation of power requirements and guided the selection of optimal production media, complementing exopolysaccharide yield data.

#### 2.8.5. Fourier Transform Infrared Spectroscopy: Attenuated Total Reflectance (FTIR-ATR)

Approximately 1–5 mg of lyophilized exopolysaccharide was recorded using FTIR-ATR (Nicolet 6700, Thermo Fisher Scientific, Waltham, MA, USA) with a resolution of 4 cm^−1^, resulting in a total of 32 scans in the wavelength range of 4000–500 cm^−1^.

## 3. Results

### 3.1. Identification of the Mushroom Strain

#### 3.1.1. Morphological Identification

*Schizophyllum commune* Fr. (MMCR00256)

**Figure 1:**
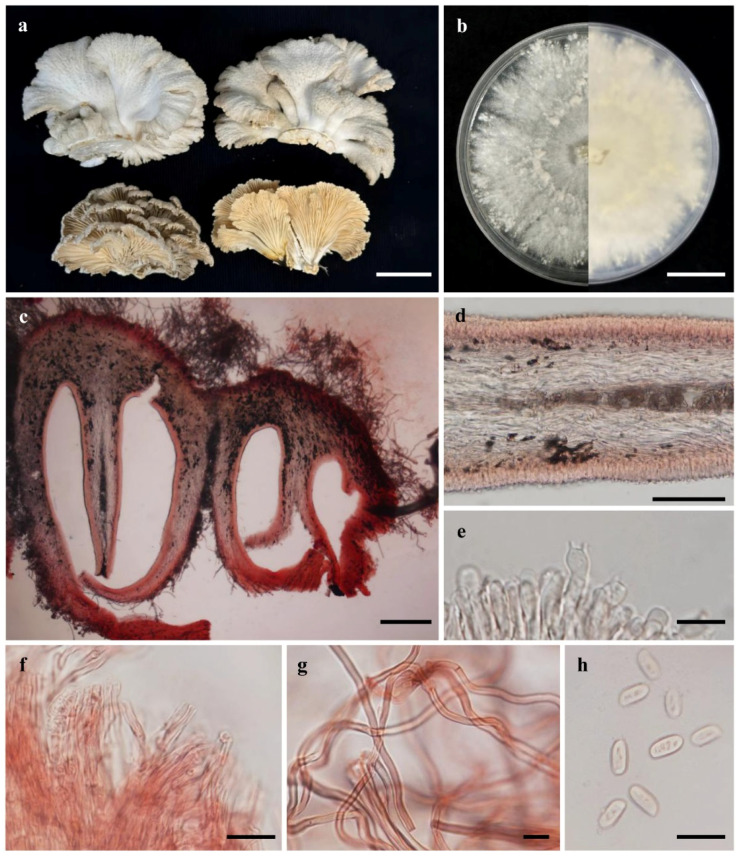
*Schizophyllum commune* Fr. (MMCR00256) (**a**) Basidiomata. (**b**) Colony on PDA (above and below). (**c**) Cross-section of the context. (**d**) Lamellar hyphae. (**e**) Basidia. (**f**) Pileipellis. (**g**) Hair hyphae. (**h**) Basidiospores. Scale bar: (**a**,**b**) = 2 cm, (**c**) = 200 μm, (**d**) = 100 μm, (**e**–**h**) = 10 μm.

Description: The pileus was 6–35 mm wide and 19–33 mm long. The flabelliform was semicircular to spatulate, sessile or semistipitate, with a whitish to grayish color. The margin was lobed, irregular, crenate, ragged, and slightly rolled toward the hymenium. The surface was covered with hairs that were frequently matted. The context (flesh) was tough, leathery, pallid, and 225–260 μm thick. The hymenium color was whitish to grayish, with split gills (lamella). The abhymenial surface was covered with hairs, hyaline, and clinging to the surface opposite that of the gills. The basidia were 15–27 × 4.5–5.0 μm (mean basidium length = 19.9 ± 3.12 μm, mean basidium width = 4.8 ± 0.25 μm, number of measured spores = 15), narrow claviform, with 2–4 sterigmata (0.5–3.0 μm long, average = 2.00 ± 0.62 μm, n = 15). Basidiospores were (5−)7.0–7.5(−8.75) × 2.5–3.0(−3.75) μm (mean spore length = 6.99 ± 0.88 μm, mean spore width = 3.01 ± 0.45 μm, number of measured spores = 30), thin- and smooth-walled, hyaline, cylindrical to subcylindrical, obliquely apiculate, and spore-printed white (Figure 1).

Specimen examined: THAILAND, Nakhon Phanom, on unidentified wood, MMCR00256 (the strain with the highest beta-glucan production).

#### 3.1.2. Phylogenetic Analysis

**Figure 2:**
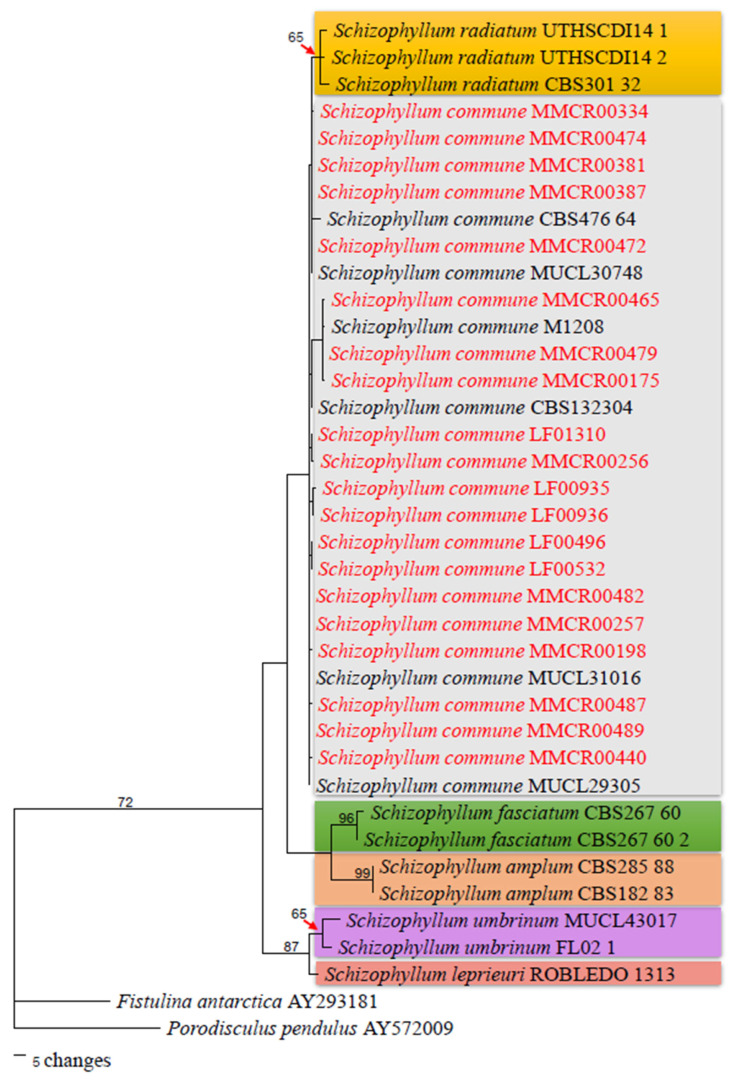
Maximum parsimony (MP) phylogenetic tree based on combined ITS and LSU rDNA sequences of the 20 fungal strains. Bootstrap values (>50%) from 1000 replicates are shown at the branches. All the strains studied, indicated in red, were clearly clustered within the *S. commune* clade.

Molecular analysis of the internal transcribed spacer (ITS) and large subunit (LSU) regions yielded DNA sequences of approximately 684 bp and 892 bp, respectively, for *S. commune*. These sequences were used to construct a phylogenetic tree using PAUP*4.0b10 (Figure 2). In this study, *Fistulina antarctica* (AY293181) and *Porodisculus pendulus* (AY572009) were used as outgroups. The results of the phylogenetic analysis revealed that all 20 strains clustered within the *S. commune* clade with high bootstrap support values (>50%), confirming their taxonomic identity as *S. commune*. This clustering is consistent with previous studies that have utilized the ITS and LSU regions for species-level identification in fungi. For example, [27]) emphasized the reliability of ITS and LSU regions as universal DNA barcodes for fungal identification at the species level, validating the selection of these strains for further research on schizophyllan production.

#### 3.1.3. Secondary Screening of High-Potential *Schizophyllum commune* Strains for Exopolysaccharide Production

Secondary screening was carried out in PDB and PYGM using a 10% (*v*/*v*) large pellet inoculum. The results are shown in Table 5. In general, most strains of *S. commune* produced less than 1 g/L exopolysaccharide in both media. However, three strains produced exopolysaccharide at relatively high yields, namely, MMCR00487 (2.67 g/L in PYGM), MMCR00474 (2.67 g/L in PDB) and MMCR00256 (2.38 g/L in PYGM). Most strains were able to produce high amounts of biomass in both media recipes. The strains with the highest yields of biomass were MMCR00487 (13.45 g/L in PDB), MMCR00256 (13.04 g/L in PDB), and MMCR00198 (13.04 g/L in PYGM). Notably, compared with the other strains, the two strains MMCR00256 and MMCR00487 produced the highest yields of exopolysaccharide and biomass. These findings are in agreement with those of previous studies reporting that different strains of *S. commune* exhibit variability in terms of schizophyllan and biomass production when cultivated in different carbon sources [28]. Therefore, three strains, MMCR00256, MMCR00474 and MMCR00487, were selected for further optimization of exopolysaccharide production using the Plackett–Burman design.

### 3.2. Media Component Optimization for Exopolysaccharide Production by S. commune

#### 3.2.1. Production of Exopolysaccharide by *S. commune* in 250 mL Flasks Using Plackett–Burman Design

The exopolysaccharide contents and biomass dry weights of the three strains of *S. commune* subjected to the 26 treatments are shown in Table 6. The use of a 10% (*v*/*v*) mycelial inoculum and Plackett–Burman design increased exopolysaccharide production to 8.34 ± 1.47 g/L by the strain MMCR00256 (treatment 11), to 4.89 ± 1.76 g/L by the strain MMCR00487 (treatment 7), and to 2.62 ± 0.01 g/L by the strain MMCR00474 (treatment 13). The strain MMCR00256 produced the greatest amount of exopolysaccharide and was selected for further optimization using the central composite design.

#### 3.2.2. Production of Exopolysaccharide by *S. commune* in 250 mL Flasks Using Central Composite Design

**Figure 3:**
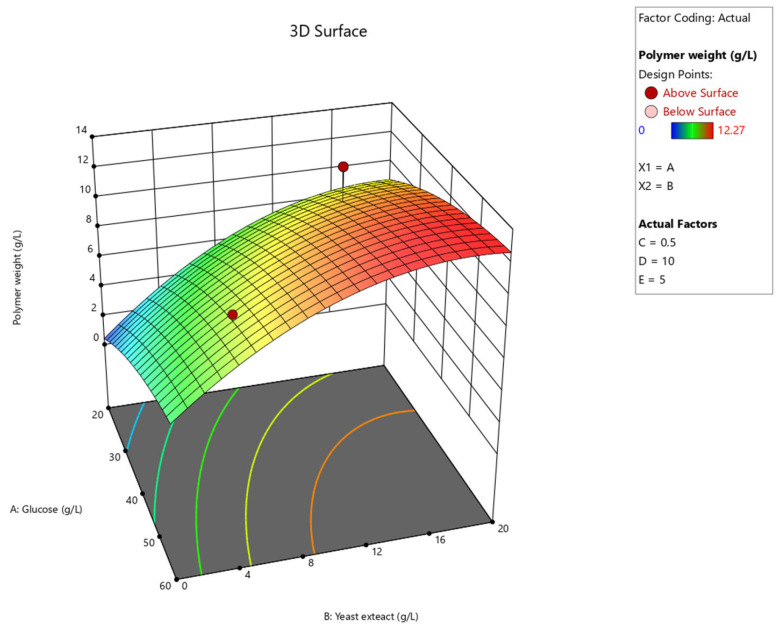
The effects of the concentrations of glucose (g/L) and yeast extract (g/L) on exopolysaccha ride production (g/L).

Five variables—glucose (A), yeast extract (B), manganese sulfate (C), glutamic acid (D) and trace elements (E)—were optimized for exopolysaccharide production by strain MMCR00256 (Table 7). The effects of glucose and yeast extract concentrations were visualized in three-dimensional response surface plots (Figure 3), which indicated that increasing the glucose to yeast extract ratio significantly increased exopolysaccharide production. The ANOVA results for the quadratic response surface model (Table 8) revealed that the overall model was significant (model F value = 8.17, *p* < 0.0001). Specifically, glucose (A), yeast extract (B), and glutamic acid (D) were identified as the most influential factors with significant effects on EPS production, whereas manganese sulfate (C) and trace elements (E) showed no significant impact within the tested ranges. The exopolysaccharide content and biomass production of the strain MMCR00256 in 250 mL flasks determined using central composite design are shown in Table 9. The results revealed that the medium containing 40 g/L of glucose, 10 g/L of yeast extract, 1.25 g/L of manganese sulfate, 12.87 g/L of glutamic acid, and 4 mL/L of the trace elements in 1 L (treatment 19) produced the highest exopolysaccharide concentration (10.39 ± 1.69 g/L), with a biomass production of 26.28 ± 1.63 g/L (395 mg EPS per g biomass) (Table 7).

### 3.3. Production of Exopolysaccharide by S. commune MMCR00256 in a 5 L Bioreactor

**Figure 4:**
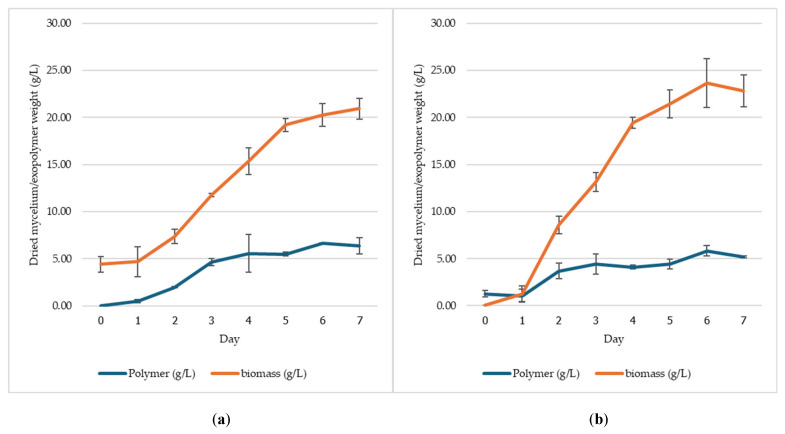
Biomass and exopolysaccharide dry weights produced by *S. commune* MMCR00256 in a 5 L bioreactor using CCD medium: mycelial seed culture (**a**) and small pellet (**b**).

**Figure 5:**
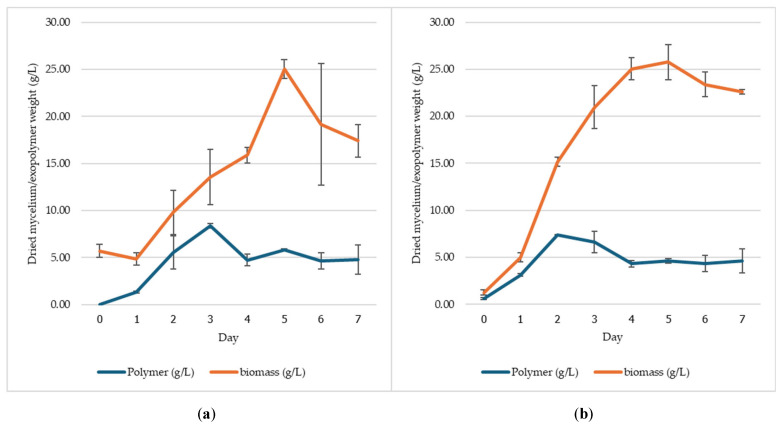
Biomass and exopolysaccharide dry weights produced by *S. commune* MMCR00256 in a 5 L bioreactor using estimated medium: mycelial seed culture (**a**) and small pellet (**b**).

The production of exopolysaccharide by *S. commune* MMCR00256 was evaluated in a 5 L bioreactor using two inoculum types (mycelial and small pellet) and two media formulations (CCD and estimated). With respect to the CCD and estimated media at agitation speeds of 100–300 rpm, the inoculum type significantly affected the exopolysaccharide yield. The CCD result using mycelial inoculum had the highest yield (6.62 g/L), and the concentration of the small pellet inoculum was 5.84 g/L on day 6 (Figure 4). In the estimated medium, the mycelial inoculum produced the highest yield of 8.37 g/L on day 3, and the lowest yield of small pellet inoculum was 7.39 g/L on day 2 (Figure 5).

Residual glucose levels were monitored daily to assess sugar consumption. In the CCD medium, the initial glucose concentration decreased to 40.0 g/L and was completely depleted by day 6. In contrast, in the estimated medium, glucose decreased more rapidly from an initial concentration of 50.0 g/L and was fully consumed by day 5. These results are consistent with the results of bioreactor production, in which the exopolysaccharide yield in the estimated medium peaked on day 3. Additionally, compared with the small pellet inoculum, the mycelial inoculum consistently produced higher exopolysaccharide yields, and compared with the CCD medium, the estimated medium yielded greater amounts of exopolysaccharide and accelerated production kinetics.

### 3.4. Exopolysaccharide Analysis

#### 3.4.1. Molecular Weight Distribution Analysis

GPC analysis of exopolysaccharide samples collected on day 7 (Table 10) revealed the molecular weight distribution in the CCD and estimated media. In the CCD medium, the chromatogram revealed three noteworthy peaks at retention times of 18.561 and 19.751 min, corresponding to a low-molecular-weight cluster (100–101 kDa), and the peak at a retention time of 11.738 min belonged to a high-molecular-weight cluster (103–104 kDa). Similarly, the estimated medium exhibited three peaks at 12.361, 19.120 and 20.317 min, which are divided into two groups of molecular weight clusters. These results demonstrate multiple peaks in the GPC chromatograms, indicating that the exopolysaccharide in each culture medium consists of high- and low-molecular-weight fractions [4]. This information is critical for guiding product development and optimizing industrial-scale production processes.

#### 3.4.2. Measurement of Exopolysaccharide Viscosity

Exopolysaccharide samples were collected from the bioreactor after 7 days of cultivation and irradiated prior to viscosity analysis using a Brookfield viscometer. The viscosity of the CCD medium was 887.25 cP, whereas that of the estimated medium was 10,755.00 cP. These results indicate a clear difference in physical properties between the two media: the lower viscosity of the CCD medium reduces the energy demands on the agitation system. In contrast, the exopolysaccharide produced in the estimated medium has a higher viscosity and requires more energy.

#### 3.4.3. FTIR-ATR Spectroscopic Analysis

**Figure 6:**
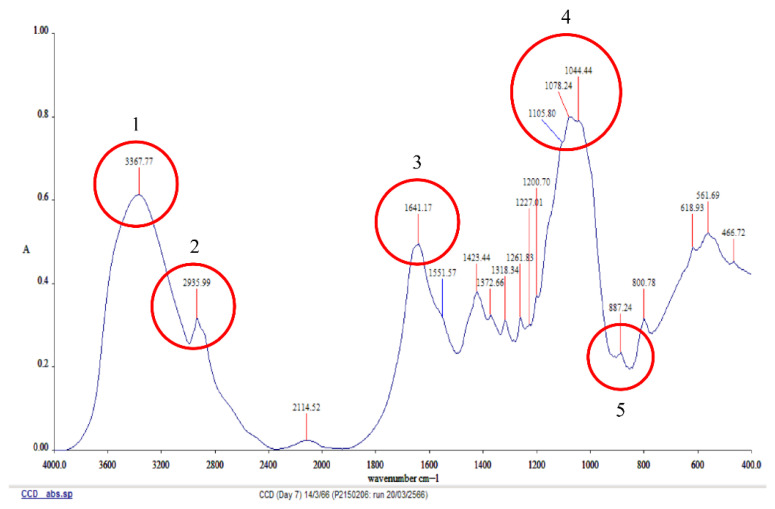
FTIR-ATR profile of exopolysaccharide by *S. commune* strain MMCR00256.

The FTIR analysis results shown in Figure 6 revealed significant absorption peaks at five points. Point 1 exhibited an absorption frequency value of 3367.71 cm^−1^, which is indicative of characteristics related to sugar groups. Point 2, at 2935.99 cm^−1^, represented C–H bond interactions. Point 3, at 1641.17 cm^−1^, corresponded to the protein region. Point 4, within the range of 1105.80–1044.44 cm^−1^, reflected the presence of sugar-related bonds. Finally, point 5, with an absorption frequency value of 887.24 cm^−1^, is consistent with the β-glycosidic linkages typically found in the structure of schizophyllan. These findings are consistent with the research conducted by Jamshidian et al. [29].

## 4. Discussion

In this study, twenty *Schizophyllum commune* isolates were collected from across Thailand and screened for schizophyllan production. Three strains of *S. commune* (MMCR00256, MMCR00474 and MMCR00487) presented the highest levels of schizophyllan. The Plackett–Burman design identified glucose, yeast extract, glutamic acid and trace elements as key factors influencing schizophyllan production, enabling MMCR00256 to reach a production of 8.34 g/L, which is consistent with the findings of [30]. Optimization using the central composite design further increased the MMCR00256 yield to 10.39 g/L (395 mg/g biomass) and scaled up the production in a 5 L bioreactor using mycelial inoculum, and the estimated medium produced schizophyllan at 8.37 g/L. The comparison between the CCD and estimated media was conducted to validate the predictive power of the statistical model during the scale-up process. While the CCD medium represented the optimal condition identified within the experimental design matrix, the estimated medium was formulated based on the model’s point prediction to explore the potential for even higher yields beyond the initial design boundaries. The results obtained from the estimated medium confirm that the statistical model employed in this study is highly effective for identifying optimal production conditions. This study differs from previous reports by Kumari et al. [21] and Li et al. [31] in that twenty *S. commune* strains collected from Thailand were systematically screened to identify strains with high potential for schizophyllan production. In addition, the effect of inoculum form on EPS production during fermentation was investigated. Furthermore, different culture media were compared at the bioreactor scale to evaluate production performance under conditions closer to practical applications. The results revealed that the amount of schizophyllan in a 5 L bioreactor was lower than that in a flask. The schizophyllan concentrations obtained in this study are similar to those reported in other studies. Mohammadi et al. [32] obtained 11.06 g/L of schizophyllan from shaken flasks and 5.77 g/L from a bioreactor in the optimization of *S. commune* (CGMCC 5.113), whereas Li et al. [31] obtained 13.95 g/L from shaken flasks and 13.68 g/L from a bioreactor in the optimization of *S. commune* (IBRC-M 30213). Furthermore, the production from bioreactors provided a yield per liter lower than that from the shaken flasks. Li et al. [31] reported that the mechanical action of the agitator might damage the mycelia and affect the production of schizophyllan. Mohammadi et al. [32] reported a negative effect of a bioreactor in which a high-speed agitator could damage the mycelia and schizophyllan production. The molecular weight of schizophyllan can vary depending on many factors, such as the production strain, culture medium, production process, and extraction process. The molecular weights of schizophyllan are as follows: native schizophyllan, 1,000,000–3,000,000 Da [33]; water-soluble form (SPG-S), 500,000–1,200,000 Da [34]; low-molecular-weight SPG, 30,000–100,000 Da [3]; acid-degraded schizophyllan, approximately 50,000 Da [35]; and gamma-irradiated schizophyllan, 10,000–70,000 Da [36]. While the FTIR-ATR and GPC results provide strong evidence of functional groups and molecular weight profiles consistent with schizophyllan, the yields reported in this study refer to the total ethanol-precipitated exopolysaccharide (EPS) fraction. Further structural elucidation, such as NMR spectroscopy, would be required to definitively confirm the branching frequency of the glucan backbone. This study revealed polysaccharide concentrations close to those of other species of mushrooms. Lee et al. [37] obtained 8.7 g/L exopolysaccharide (EPS) from the mushroom *Grifola frondosa*, whereas Wu et al. [38] obtained 5.3 g/L EPS from *Auricularia auricular*. However, the beta-glucan production from mushrooms was lower than that from insect fungi. Prathumpai et al. [39] obtained 35.77 g/L of beta-glucan from shaken flasks and 76.87 g/L from a bioreactor by the insect fungus *Ophiocordyceps dipterogena* (BCC2073). Morphological examination of the *S. commune* isolates revealed typical macroscopic features: white to gray coloration, fan-shaped caps with lobed margins and distinctive split gills. Microscopic characterization further confirmed classical cellular structures such as narrow claviform basidia and cylindrical to subcylindrical basidiospores, which aligned with established morphological descriptions [19]. Moreover, phylogenetic analysis demonstrated that all twenty isolates clustered clearly within the *S. commune* clade (bootstrap support > 50%) [27]. This study demonstrates that *S. commune* strain MMCR00256 from Thailand is a highly promising candidate for schizophyllan production. Utilizing the estimated medium in combination with a mycelial inoculum achieved the peak yield within only 3 days, which was significantly faster than the CCD medium. This reduction in cultivation time is vital for industrial-scale production, as it minimizes energy and labor costs while increasing annual throughput key drivers of commercial viability. Future research will focus on in-depth bioreactor optimization and detailed molecular characterization using various techniques to definitively confirm the schizophyllan structure.

## Figures and Tables

**Table 1:** List of *S. commune* fungal isolates used for exopolysaccharide screening.

No.	Original Code	BCC Code	Substrate of Isolation	Province
1	LF00496	61961	*Bambusa* sp.	Chiang Rai
2	LF00532	61997	*Bambusa* sp.	Chiang Rai
3	LF00935	64067	*Bambusa* sp.	Chiang Rai
4	LF00936	64068	*Bambusa* sp.	Chiang Rai
5	LF01310	73957	*Bambusa* sp.	Si Sa ket
6	MMCR00175	-	Wood (Unidentified)	Pathum Thani
7	MMCR00198	91347	Wood (Unidentified)	Chiang Mai
8	MMCR00256	92766	Wood (Unidentified)	Nakhon Phanom
9	MMCR00257	92768	Wood (Unidentified)	Nonthaburi
10	MMCR00334	93326	Rachis (*Elaeis guineensis*)	Trang
11	MMCR00381	94275	Branch (*Hevea brasiliensis*)	Trang
12	MMCR00387	94280	Trunk (*Tamarindus indica*)	Kalasin
13	MMCR00440	94317	Branch (*H. brasiliensis*)	Nakhon Phanom
14	MMCR00465	94321	Wood (Unidentified)	Nonthaburi
15	MMCR00472	94326	Branch (*Parkia speciosa*)	Trang
16	MMCR00474	94286	Branch (*H. brasiliensis*)	Trang
17	MMCR00479	94291	Wood (Unidentified)	Nonthaburi
18	MMCR00482	94292	Rachis (*Elaeis guineensis*)	Chumphon
19	MMCR00487	94295	Trunk (*Cocos nucifera*)	Pathum Thani
20	MMCR00489	94368	*Bambusa* sp.	Bangkok

**Table 2:** Different influential factors used for exopolysaccharide production by *S. commune* at minimum and maximum levels in the Plackett–Burman design.

Factor	Chemical	Units	Minimum	Maximum
A	Glucose	g/L	20.00	40.00
B	Xylose	g/L	0.00	5.00
C	Mannose	g/L	0.00	5.00
D	Diammonium hydrogen phosphate	g/L	10.00	20.00
E	Yeast extract	g/L	0.00	5.00
F	Casein hydrolysate	g/L	0.00	5.00
G	Peptone	g/L	0.00	5.00
H	Manganese sulfate	g/L	0.00	0.50
I	Glutamic acid	g/L	0.00	3.00
J	Trace elements *	mL/L	1.00	3.00
K	Vitamin solution	mL/L	1.00	3.00

* The trace elements consisted of zinc sulfate (14.30 g/L), iron sulfate (13.80 g/L), manganese chloride (6.00 g/L), and copper sulfate (2.50 g/L).

**Table 3:** Variables selected for the 26 treatments with different media compositions using the Plackett–Burman design.

No.	Factors										
	A	B	C	D	E	F	G	H	I	J	K
	g/L	g/L	g/L	g/L	g/L	g/L	g/L	g/L	g/L	mL/L	mL/L
1	40	5	0	20	5	5	0	0	0	3	1
2	20	5	5	10	5	5	5	0	0	1	3
3	40	0	5	20	0	5	5	0.5	0	1	1
4	20	5	0	20	5	0	5	0.5	3	1	1
5	20	0	5	10	5	5	0	0.5	3	3	1
6	20	0	0	20	0	5	5	0	3	3	3
7	40	0	0	10	5	0	5	0.5	0	3	3
8	40	5	0	10	0	5	0	0.5	3	1	3
9	40	5	5	10	0	0	5	0	3	3	1
10	20	5	5	20	0	0	0	0.5	0	3	3
11	40	0	5	20	5	0	0	0	3	1	3
12	20	0	0	10	0	0	0	0	0	1	1
13	30	2.5	2.5	15	2.5	2.5	2.5	0.25	1.5	2	2
14	20	0	5	10	0	0	5	0.5	3	1	3
15	40	0	0	20	0	0	0	0.5	3	3	1
16	20	5	0	10	5	0	0	0	3	3	3
17	40	0	5	10	0	5	0	0	0	3	3
18	40	5	0	20	0	0	5	0	0	1	3
19	40	5	5	10	5	0	0	0.5	0	1	1
20	20	5	5	20	0	5	0	0	3	1	1
21	20	0	5	20	5	0	5	0	0	3	1
22	20	0	0	20	5	5	0	0.5	0	1	3
23	40	0	0	10	5	5	5	0	3	1	1
24	20	5	0	10	0	5	5	0.5	0	3	1
25	40	5	5	20	5	5	5	0.5	3	3	3
26	30	2.5	2.5	15	2.5	2.5	2.5	0.25	1.5	2	2

Remarks: A = glucose, B = xylose, C = mannose, D = (NH_4_)_2_HPO_4_, E = yeast extract, F = casein hydrolysate, G = peptone, H = MnSO_4_, I = glutamic acid, J = trace elements, K = vitamin solution.

**Table 4:** Composition of the estimated formulation used in a 5 L bioreactor.

Chemical	Units	Concentration
Glucose	g/L	50.00
Yeast extract	g/L	15.00
Manganese sulfate	g/L	0.85
Glutamic acid	g/L	10.00
Potassium dihydrogen phosphate	g/L	0.50
Diammonium hydrogen phosphate	g/L	0.20
Magnesium sulfate	g/L	0.20
Trace elements	mL/L	5.00
Vitamin solution	mL/L	1.00

The trace elements consist of zinc sulfate (14.30 g/L), iron sulfate (13.80 g/L), manganese chloride (6.00 g/L), copper sulfate (2.50 g/L), and nickel chloride (0.50 g/L).

**Table 5:** Exopolysaccharide and biomass production of *S. commune* using PDB and PYGM.

No	Code	Scientific Names	EPS Dried Weight (g/L)		Biomass Dried Weight (g/L)	
			PDB	PYGM	PDB	PYGM
1	LF00496	*S. commune*	0.22 ± 0.05	0.39 ± 0.03	8.19 ± 2.64	9.52 ± 0.48
2	LF00532	*S. commune*	0.34 ± 0.04	1.05 ± 0.11	8.19 ± 0.51	12.71 ± 0.13
3	LF00935	*S. commune*	0.90 ± 0.07	1.46 ± 0.20	9.37 ± 0.24	12.13 ± 1.46
4	LF00936	*S. commune*	0.28 ± 0.13	0.24 ± 0.07	7.59 ± 0.90	9.18 ± 2.01
5	LF01310	*S. commune*	0.18 ± 0.03	0.78 ± 0.18	6.55 ± 0.76	9.90 ± 0.46
6	MMCR00175	*S. commune*	0.44 ± 0.08	0.70 ± 0.15	12.25 ± 1.38	9.78 ± 0.70
7	MMCR00198	*S. commune*	0.80 ± 0.08	0.58 ± 0.05	10.10 ± 0.45	13.04 ± 0.24
8	MMCR00256	*S. commune*	1.13 ± 0.07	2.38 ± 0.08	13.04 ± 0.18	10.86 ± 0.48
9	MMCR00257	*S. commune*	1.54 ± 0.58	1.83 ± 0.10	8.98 ± 0.89	12.25 ± 0.14
10	MMCR00334	*S. commune*	2.19 ± 0.38	1.38 ± 0.34	12.79 ± 0.09	11.18 ± 1.18
11	MMCR00381	*S. commune*	0.90 ± 0.05	1.02 ± 0.26	8.41 ± 0.65	11.19 ± 1.46
12	MMCR00387	*S. commune*	0.66 ± 0.02	0.85 ± 0.39	9.02 ± 0.24	10.31 ± 1.06
13	MMCR00440	*S. commune*	0.48 ± 0.02	1.10 ± 0.11	11.49 ± 0.08	9.81 ± 0.40
14	MMCR00465	*S. commune*	0.95 ± 0.59	1.07 ± 0.67	10.16 ± 0.45	12.09 ± 1.54
15	MMCR00472	*S. commune*	0.88 ± 0.06	0.70 ± 0.10	10.15 ± 0.35	10.56 ± 1.14
16	MMCR00474	*S. commune*	2.67 ± 0.11	0.99 ± 0.12	10.47 ± 0.22	9.63 ± 0.03
17	MMCR00479	*S. commune*	0.72 ± 0.26	0.98 ± 0.09	10.00 ± 1.26	10.76 ± 1.18
18	MMCR00482	*S. commune*	1.81 ± 0.14	1.36 ± 0.28	11.65 ± 1.58	12.17 ± 0.40
19	MMCR00487	*S. commune*	1.35 ± 0.07	2.67 ± 0.25	13.45 ± 0.06	12.99 ± 0.30
20	MMCR00489	*S. commune*	0.27 ± 0.12	0.15 ± 0.18	10.25 ± 0.79	8.91 ± 0.74

**Table 6:** Exopolysaccharide and biomass production of 3 strains of *S. commune* (MMCR00256, MMCR00474, and MMCR00487) using Plackett–Burman design.

Treatment	*S. commune* (MMCR00256)		*S. commune* (MMCR00474)		*S. commune* (MMCR00487)	
	Biomass	EPS	Biomass	EPS	Biomass	EPS
	Dried Weight	Dried Weight	Dried Weight	Dried Weight	Dried Weight	Dried Weight
	(g/L)	(g/L)	(g/L)	(g/L)	(g/L)	(g/L)
1	18.31 ± 3.38	7.97 ± 1.05	21.64 ± 3.09	1.08 ± 0.60	12.79 ± 3.45	1.81 ± 0.72
2	14.49 ± 10.19	3.72 ± 4.58	18.27 ± 0.00	1.24 ± 0.44	20.69 ± 0.26	1.49 ± 0.70
3	5.42 ± 0.96	2.12 ± 0.41	13.29 ± 0.56	1.18 ± 0.16	1.93 ± 0.40	0.75 ± 0.51
4	10.74 ± 5.21	3.92 ± 2.40	11.38 ± 0.51	2.32 ± 0.83	2.03 ± 0.02	1.09 ± 0.80
5	17.20 ± 0.72	6.18 ± 0.18	13.45 ± 0.20	1.70 ± 0.42	17.09 ± 0.48	2.34 ± 0.37
6	8.42 ± 4.14	5.25 ± 2.87	8.18 ± 0.38	1.17 ± 0.30	3.66 ± 0.00	1.01 ± 0.00
7	25.71 ± 1.08	4.74 ± 1.51	20.99 ± 0.39	0.82 ± 0.32	30.07 ± 1.46	4.89 ± 1.76
8	24.79 ± 1.15	6.13 ± 0.36	23.56 ± 0.05	1.35 ± 0.28	22.02 ± 2.52	2.39 ± 0.41
9	9.05 ± 5.68	2.50 ± 0.64	22.72 ± 0.23	0.00 ± 0.00	21.27 ± 1.59	2.01 ± 0.52
10	9.89 ± 0.60	4.72 ± 2.20	12.86 ± 0.77	1.83 ± 0.44	1.79 ± 0.16	0.70 ± 0.51
11	22.36 ± 1.35	8.34 ± 1.47	21.41 ± 0.69	2.54 ± 1.27	18.50 ± 1.18	3.73 ± 1.58
12	9.55 ± 0.13	3.21 ± 0.84	8.39 ± 0.13	0.39 ± 0.53	10.14 ± 0.15	0.78 ± 0.09
13	17.19 ± 0.10	3.72 ± 2.61	17.84 ± 0.64	2.62 ± 0.01	14.64 ± 2.90	3.04 ± 1.44
14	13.82 ± 1.33	3.84 ± 0.87	13.15 ± 0.13	2.01 ± 0.83	13.84 ± 0.09	1.44 ± 0.37
15	16.19 ± 1.89	7.70 ± 0.32	18.56 ± 1.13	1.73 ± 0.19	1.90 ± 0.62	1.00 ± 0.06
16	14.97 ± 0.91	6.48 ± 1.20	12.56 ± 1.66	1.21 ± 0.05	18.30 ± 0.52	2.09 ± 0.90
17	22.94 ± 0.31	6.10 ± 0.85	18.85 ± 0.03	0.01 ± 0.00	23.58 ± 0.06	2.36 ± 0.52
18	20.29 ± 0.39	6.94 ± 0.67	19.69 ± 1.70	0.98 ± 0.14	2.29 ± 0.04	0.93 ± 0.75
19	22.91 ± 7.71	5.57 ± 2.63	21.10 ± 2.09	0.49 ± 0.00	25.71 ± 0.63	2.85 ± 0.57
20	11.54 ± 1.21	4.92 ± 4.60	12.02 ± 0.19	1.03 ± 0.62	4.20 ± 3.15	0.84 ± 0.12
21	13.78 ± 2.85	6.93 ± 2.40	11.11 ± 0.20	1.03 ± 0.32	7.14 ± 5.45	2.37 ± 1.84
22	11.59 ± 2.16	4.94 ± 2.48	8.17 ± 0.35	0.73 ± 0.40	1.92 ± 0.13	0.47 ± 0.16
23	11.61 ± 7.65	4.21 ± 0.94	21.07 ± 0.46	1.55 ± 0.77	27.88 ± 2.66	2.82 ± 0.82
24	17.55 ± 3.54	2.38 ± 1.05	13.93 ± 0.58	1.30 ± 0.21	14.18 ± 1.88	1.08 ± 0.11
25	12.96 ± 9.29	3.34 ± 2.17	22.76 ± 1.05	1.19 ± 0.22	2.07 ± 0.36	0.74 ± 0.32
26	20.07 ± 2.31	6.26 ± 1.27	17.31 ± 1.70	1.99 ± 0.70	11.57 ± 0.00	0.64 ± 0.00
PDB	9.87 ± 0.53	2.91 ± 0.51	10.88 ± 0.14	0.57 ± 0.06	3.26 ± 0.44	0.71 ± 0.04
PYGM	9.02 ± 0.80	4.26 ± 0.77	7.93 ± 0.23	0.80 ± 0.14	11.18 ± 1.07	2.29 ± 0.30

**Table 7:** Different parameters affected exopolysaccharide production by *S. commune* strain MMCR00256 using central composite design.

Factor	Chemical	Units	Minimum	Maximum
A	Glucose	g/L	21.79	58.21
B	Yeast extract	g/L	0.8942	19.11
C	Manganese sulfate	g/L	−0.1159	2.62
D	Glutamic acid	g/L	0.1259	12.87
E	Trace element	mL/L	2.18	5.82

**Table 8:** ANOVA for exopolysaccharide production by *S. commune* MMCR00256.

Source	Sum of Squares	df	Mean Square	F Value	*p* Value	
Model	340.35	20	17.02	8.17	<0.0001	significant
A—Glucose	13.89	1	13.89	6.67	0.0131	
B—Yeast extract	61.63	1	61.63	29.58	<0.0001	
C—Manganese sulfate	8.10	1	8.10	3.89	0.0548	
D—Glutamic acid	52.81	1	52.81	25.35	<0.0001	
E—Trace element	0.7633	1	0.7633	0.3664	0.5480	

**Table 9:** Exopolysaccharide and biomass dried weight produced by *S. commune* strain MMCR00256 using central composite design.

Treatments	Factor/Chemical					Production	
	(A) Glucose	(B) Yeast Extract	(C) Manganese Sulfate	(D) Glutamic Acid	(E) Trace Elements	Exopolysaccharide Dried Weight (g/L)	Biomass Dried Weight (g/L)
1	50.00	15.00	0.50	10.00	3.00	8.52 ± 0.35	28.32 ± 3.60
2	50.00	5.00	2.00	10.00	3.00	5.48 ± 1.64	18.29 ± 4.08
3	30.00	15.00	2.00	3.00	5.00	3.53 ± 1.86	17.95 ± 4.55
4	50.00	15.00	2.00	3.00	3.00	6.88 ± 0.46	26.25 ± 2.11
5	50.00	15.00	0.50	3.00	5.00	6.00 ± 0.83	25.20 ± 4.66
6	50.00	5.00	0.50	10.00	5.00	8.17 ± 0.48	25.83 ± 0.42
7	30.00	5.00	2.00	10.00	5.00	4.28 ± 0.48	14.51 ± 2.70
8	30.00	15.00	0.50	10.00	5.00	9.90 ± 2.15	23.44 ± 4.03
9	50.00	5.00	2.00	3.00	5.00	1.91 ± 0.27	12.91 ± 0.77
10	30.00	15.00	2.00	10.00	3.00	6.05 ± 3.88	17.78 ± 7.38
11	30.00	5.00	0.50	3.00	3.00	4.52 ± 0.96	19.07 ± 1.31
12	21.79	10.00	1.25	6.50	4.00	4.57 ± 1.15	16.59 ± 0.54
13	58.21	10.00	1.25	6.50	4.00	7.62 ± 0.73	28.07 ± 2.78
14	40.00	0.89	1.25	6.50	4.00	1.61 ± 0.08	10.22 ± 1.90
15	40.00	19.11	1.25	6.50	4.00	8.02 ± 0.91	25.79 ± 0.42
16	40.00	10.00	−0.12	6.50	4.00	7.09 ± 0.77	19.55 ± 0.54
17	40.00	10.00	2.62	6.50	4.00	4.77 ± 1.03	16.77 ± 5.87
18	40.00	10.00	1.25	0.13	4.00	4.46 ± 0.78	20.56 ± 0.63
19	40.00	10.00	1.25	12.87	4.00	10.39 ± 1.69	26.28 ± 1.63
20	40.00	10.00	1.25	6.50	2.18	7.06 ± 0.74	22.84 ± 0.67
21	40.00	10.00	1.25	6.50	5.82	6.35 ± 0.48	21.57 ± 2.97
22	40.00	10.00	1.25	6.50	4.00	5.94 ± 0.54	22.56 ± 4.19
PDB	-	-	-	-	-	2.53 ± 0.13	13.47 ± 0.50
PYGM	-	-	-	-	-	5.38 ± 0.21	9.27 ± 0.12

**Table 10:** Molecular weight distribution of schizophyllan determined by GPC.

Medium Type	Cultivation Time (Days)	Molecular Weight Distribution (%)	
		10^3^–10^4^ (kDa)	10^0^–10^1^ (kDa)
CCD	7	4.33	95.67
ES	7	11.99	88.01

## Data Availability

The original contributions presented in this study are included in the article. Further inquiries can be directed to the corresponding authors.
